# Melanin in the Retinal Epithelium and Magnetic Sensing: A Review of Current Studies

**DOI:** 10.3390/biophysica4040030

**Published:** 2024-09-25

**Authors:** Lidia Zueva, Vassiliy Tsytsarev, Janaina Alves, Mikhail Inyushin

**Affiliations:** 1 Department of Microbiology and Immunology, Universidad Central del Caribe, Bayamon, PR 00960, USA; 2 Department of Neurobiology, University of Maryland School of Medicine, Baltimore, MD 21201, USA

**Keywords:** melanin, magnetic sensitivity, cryptochrome

## Abstract

Coming in a variety of forms, melanin is one of the most abundant, stable, diverse, and evolutionarily ancient pigments found in living things in nature. These pigments often serve protective functions, typically well-adapted to their specific roles. One such protective function is metal chelation and cation exchange, which help regulate and buffer metal concentrations within cells. By binding to certain metals, melanin can acquire magnetic properties. Because of this, it may play a role in magnetic effects and possibly in the response of organisms to external magnetic fields and magnetic sensing. While there is melanin in plants, microbes, fungi, and invertebrates, certain types of melanin are specifically associated with the retina in vertebrates, including migrating bird and fish species. In this review, we examine studies focusing on the properties of melanin in these parts of the body and their possible association with magnetic sensing, and generally, magnetic sensing in the retina.

## 1. Introduction

Melanin in vertebrates: Melanin variants are natural protective polymers formed through the oxidative polymerization of L-dopa, derived from the oxidation of tyrosine [1–3]. While bearing the same name, melanin types can be different and very specific in their functions. In vertebrates, melanin is present in the skin (including hair fur or plumes), inner ear, eyes, and brain.

Melanin in the skin, hair, or plumes is present in two main types: eumelanin (black or brown melanin), which is mainly composed of oligomers of dihydroxy indole and its derivatives, and pheomelanin (yellow or orange–red melanin), which is derived from benzothiazine units, and these melanin types determine the shades of black and yellow in hair [3]. In the brain, neuromelanin is ubiquitous but especially concentrated in catecholaminergic neurons of the substantia nigra and locus coeruleus. In humans, catecholaminergic neurons are characterized by an age-related accumulation of neuromelanin (NM), rendering the soma of the neurons dark. This intracellular NM appears to serve as a very efficient quencher for toxic molecules that may bond and eliminate neurotoxins, and even sequester and release dopamine that may help regulate dopamine levels, particularly during periods of high neuronal activity when dopamine can become overloaded [4,5]. However, rodents generally lack neuromelanin, and mice are completely devoid of NM, but rats seem to form a small amount at an older age [6]. Human substantia nigra neuromelanin is composed of two different forms of neuromelanin—pheomelanin and eumelanin—in a specific double-membrane organelle that can be observed with transmission electron microscopy, where pheomelanin is in the core of these organelles surrounded by eumelanin [7]. Neuromelanin is also described as a multilayer structure with planar overlapped sheets consisting of dihydroxy indole and benzothiazine rings and additional unidentified groups, stacked much higher in NM than in other synthetic or naturally occurring melanin [8]. Neuromelanin in the substantia nigra (SN) may reflect dopamine oxidation, and since after adhesion to iron can be quantified using neuromelanin-sensitive magnetic resonance imaging (neuromelanin-MRI) in a non-invasive manner in humans, there is great promise for using it as a biomarker associated with dopamine disorders.

In the inner ear, melanin pigment, which includes both eumelanin and pheomelanin, is normally present adjacent to capillaries in the stria vascularis near hair cells and vestibular organs. Melanin in the inner ear has been linked to hearing function. People with too little melanin are more susceptible to noise damage and have poorer sound localization abilities [9]. Deafness in one or both ears is often associated with blue eyes and white pigmentation [10]. Black individuals have a lower risk of hearing loss than whites, possibly because of differences in cochlear melanocytes [11].

In the context of the meninges, melanin produced by meningeal melanocytes has been observed in various species, including humans, and is primarily located in the leptomeninges, which are the two inner layers of the meninges, namely, the arachnoid mater and pia mater. They are typically found in isolated clusters rather than uniformly distributed across the meninges [12,13]. While the exact type of melanin in meningeal melanocytes is not explicitly detailed in the available literature, it is reasonable to infer that dark eumelanin is predominant, as it is the most common color tone of meninges according to our experience with brain surgery in small animals.

In the eye: It is known that both eumelanin and pheomelanin are present in the iridal and choroidal melanocytes (so-called uveal zone) in many vertebrates, including humans [14]. The quantity and type of melanin in the uveal melanocytes determine the color of the iris. Also, melanocytes are located in the retina within the layer of retinal pigment epithelium cells.

## 2. Retinal Pigment Epithelium (RPE)

The retinal pigment epithelium contains mostly eumelanin, which can be brown or black [15,16]. Both black and brown eumelanin are primarily composed of two key molecules: 5,6-dihydroxyindole (DHI) and 5,6-dihydroxyindole-2-carboxylic acid (DHICA). Both molecules are derived from the oxidation of the amino acid tyrosine, which is catalyzed by the enzyme tyrosinase during the process of melanogenesis. 5,6-Dihydroxyindole (DHI) contributes to the darker shades of eumelanin, resulting in black pigmentation, and 5,6-Dihydroxyindole-2-carboxylic acid (DHICA) is associated with lighter shades of eumelanin, contributing to brown–reddish pigmentation [17].

Both DHI and DHICA undergo oxidation and polymerization to form eumelanin. Under natural conditions, these two components often co-polymerize, leading to a range of eumelanin polymers that vary in color and solubility. The ratio of DHI to DHICA can influence the properties of the resulting eumelanin, including its color intensity and solubility characteristics. Overall, the synthesis of dark eumelanin involves a complex interplay of these molecules, resulting in the diverse pigmentation observed in RPE.

**Figure 1.**
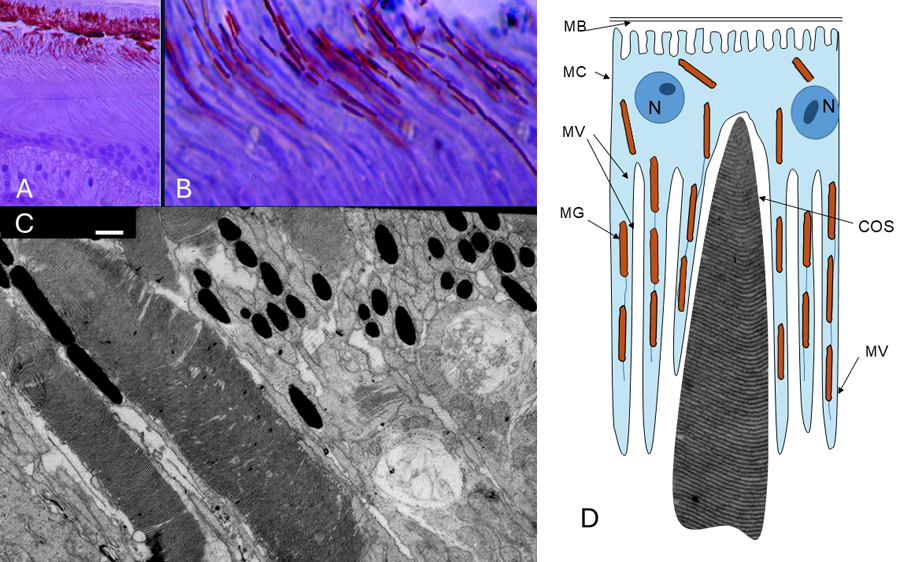
(**A**) Slice of bird retina (Ficedula hipoleuca) stained with toluidine blue, showing the retinal pigment epithelium (RPE) layer (brown–red) between other layers. (**B**) Part of Figure (**A**), showing the RPE microvilli with the train of melanosomes (brown–red) inside the microvilli. (**C**) Electron microphotography of melanosomes (black) of the same bird species between outer segments of cones (left part of the image), and also showing cut melanosomes inside processes (microvilli) of RPE cells surrounding other cones (right part of the image). (**D**) Schematic of an RPE melanocyte (MC) and microvilli (MV), which contain the chains of melanin granules, melanosomes (MG). Note, that microvilli extend along practically the entire length of the cone cell outer segment (COS). MB—Bruch membrane. Bar—100 microns in (**A**), 10 microns in (**B**), and 0.5 microns in (**C**). A description of methods used to produce these images can be found in [26,27].

The Retinal Pigment Epithelium (RPE) is naturally intensely pigmented, with melanin granules (melanosomes) presented in the cytoplasm of the RPE cells, and their processes. Melanosomes in RPE are specialized organelles of elongated form (Figure 1B), while in transversally cut electron microscopic images, they may look circular or oval in shape (Figure 1C). Interestingly, the color of melanin in the Ficedula hipoleuca bird species is brown–red (Figure 1A,B), suggesting that eumelanin contains many DHICA molecules. In retinal pigment epithelium, most melanosome formation occurs during embryonic development, with limited new melanosome production in adulthood [18]. Being the optical anti-reflective coating for reducing unnecessary lateral glare, melanin in the RPE cells also serves as a photoprotector, absorbing excess light from the photoreceptors and protecting the retina from photo-oxidation and oxidative stress [19,20]. Microvilli (processes of RPE cells with melanosomes) extend from the apical surface of the RPE cells to envelop the outer segments of both rods and cones (see also Figure 1B), although the length of the outer segment cloaked by the microvilli is greater in rods than cones [21].

In mammals, melanosomes travel bi-directionally along microtubule tracks. The anterograde movement, i.e., towards microtubule plus-ends at the periphery, is accomplished by proteins in the kinesin superfamily, whereas the retrograde movement, i.e., towards microtubule minus-ends at the cell center, is achieved by dynein and dynein-associated proteins [22]. With actin filaments, melanosomes inside the microvilli are interconnected, forming a chain, small GTPase Rab27a links myosin VIIa (molecular motor) to the melanosome surface, constraining the organelles within a zone of filamentous actin [23]. The whole chain of melanosomes can be retracted from the process inside the RPE cell body or pushed back into the process, depending on light conditions (called retinomotor reaction). This train of melanosomes will either optically separate one cone from another, forming an optical border, or, being retracted, remove the optical border between cones. Optical separation of cones augments the optical resolution of the retina, while the retraction of melanosomes from processes reduces optical resolution. Because RPE cells retract melanosomes from microvilli under low light conditions, it reduces the optical resolution of the retina in this condition, making a kind of pixel binning in modern video cameras and augmenting sensitivity to light. During retinomotor reaction, besides changes inside RPE microvilli, there are changes in the length of different cones and rods. The whole process is an important part of the retinal light adaptation mechanism [19,24].

The amount of melanin in the RPE decreases significantly with age, and melanin biosynthesis is minimal or absent in adult human RPE cells [15,25]. The gradual loss of RPE melanin compromises its photoprotective mechanism, indicates ocular senescence, and makes the eye susceptible to degenerative diseases like age-related macular degeneration (AMD) [15].

Melanin participates in the regulation of iron in RPE, and melanosomes were shown to protect ARPE-19 cells, a human retinal pigment epithelium (RPE) cell line, against oxidative stress [28]. Iron dysregulation in conjunction with other disease processes may exacerbate retinal degeneration. It was shown that long-term iron ion overload produces clear macular degeneration due to ferroptosis [29]. There is an opposite effect: research indicates that iron accumulation within RPE cells significantly influences melanin content. In studies involving human fetal RPE cells and ARPE-19 cells, treatment with iron in the form of ferric ammonium citrate resulted in increased pigmentation and melanosome numbers, suggesting a direct relationship between iron levels and melanin synthesis [30]. The studies also indicate that age-related changes in RPE cells can affect both melanin and iron levels, with older cells exhibiting a decline in melanin content and potentially altered iron homeostasis [28].

This raises the issue of the magnetic properties of melanin and its complexes.

## 3. Bounding of Metals to Melanin and Its Magnetic Properties

Eumelanin (the main melanin in RPE) is a complex biopolymer known for its ability to scavenge metals, lipids, and peptides [31]. The binding of metals is of special interest. Research has shown that melanin possesses a high affinity and binding capacity for metal ions, and natural melanin also contains a wide variety of bound metals, including potassium, sodium, calcium iron, copper, manganese, and zinc [32–34]. Interestingly, the binding affinity of melanin for various metals varies. It has been reported that heavy metals such as Fe(III), Cu(II), Pb(II), and La(III) exhibit a stronger binding affinity compared to lighter metals. Additionally, melanin can act as a reservoir for essential ions like Ca(II) and Zn(II), which can be released under specific physiological conditions. Furthermore, the iron-binding capacities have been evaluated, indicating the ability of melanin to scavenge paramagnetic metals, including Fe(III) iron. The interaction between melanin and metal ions is influenced by pH, with different metal ions showing varying degrees of binding capacity depending on the environmental conditions [33]. The binding of iron to melanin has been studied using techniques such as magnetometry, electron paramagnetic resonance, and nuclear magnetic relaxation dispersion, revealing the complexities of the magnetic behavior of iron-loaded melanin and its dependence on pH, but confirming that natural melanin can be paramagnetic [32,35]. Melanin can exhibit superparamagnetic properties when complexed with Fe(III) ions. Studies indicate that while some iron ions may couple antiferromagnetically, others can remain uncoupled, exhibiting superparamagnetic behavior [36]. This balance can lead to overall superparamagnetism under certain conditions, especially when the size of the iron cluster is small enough to avoid magnetic ordering [37].

Overall magnetic properties of metallomelanin complex molecules: Melanin polymers consisting of tyrosine derivatives alone at normal temperature displayed little magnetic susceptibility (as low as 4.42 × 10^–7^ emu g^–1^ Oe^–1^), indicating that the natural melanin polymer is magnetically inactive. However, melanin can bind various metal ions, including iron, and the interaction between melanin and metal ions can modulate their properties, so, synthetic melanin loaded with magnetic iron can be used to mechanically control cells with a magnetic field [38]. Similarly, synthetic melanin can be used to purify water from heavy metals as a metal scavenger [39] and to mechanically remove bacteria and plastic fragments (melanin can be very steaky) with an external magnetic field [40,41]. Additionally, the interaction between melanin and iron has been investigated through power saturation studies of the electron paramagnetic resonance spectra, which determined the relative amount of paramagnetic metals bound by melanin, and the possibility of using its paramagnetism for magnetic resonance imaging (MRI) [42]. Therefore, while melanin itself is not inherently magnetic, its interaction with iron and other metal ions can modulate its magnetic properties. Interestingly, substantial amounts of diverse metal ions are usually bound to the two types of melanosomes. Cu(II) and Zn(II) are present in similar amounts, while the Fe(III) content is four times higher in the reddish melanosomes, and may reach 1.6 mmol/g or more [33,43]. Melanin in bird RPE is brown–reddish and thus contains many DHICA molecules (see Figure 1A,B). Research indicates that DHICA has stronger Fe(III) chelation properties than 5,6-dihydroxyindole (DHI). This is evidenced by the higher binding constants observed for DHICA in spectrophotometric titrations, which measure the strength of the interaction between DHICA and Fe(III) [44]. The paramagnetic properties of melanin can be used for melanin detection. For example, in neurodegenerative pathologies characterized by selective loss of catecholaminergic neurons, such as PD, the levels of neuromelanin are reduced [8]. Loss of neurons containing neuromelanin in humans may be considered a hallmark of Parkinson’s disease (PD). At the presynaptic terminal of dopamine neurons, NM plays a key role in the development of neurodegenerative disorders, including PD [5]. There is a hypothesis that neuromelanin is involved in the mechanism of presynaptic vesicular transport of dopamine. This biological mechanism may be the basis for memory formation in dopamine neurons [45]. The paramagnetic properties of neuromelanin-bound iron and, to a lesser extent, copper, show that these brain areas contain neuromelanin hyperintense on T1 gradient echo MRI [45]. The amount of melanin in substancia nigra can be detected by magnetic resonance, accurately detecting Parkinson’s disease [46,47]. Magnetic resonance can be used for melanoma detection (paramagnetic melanin); even in persons with black skin, the amount of paramagnetic melanin in melanoma is greater [48].

Paramagnetic materials have a permanent dipole moment or permanent magnetic moment and are weakly attracted to external magnetic fields. Because the net magnetic moment is aligned with the field, it produces a force. Paramagnetic particles are attracted to regions of higher magnetic field strength. When subjected to an external magnetic field, these particles experience a force that can cause them to move toward the source of the field [49]. It is known that paramagnetic materials are primarily responsive to static or slowly varying magnetic fields rather than alternating magnetic fields. Paramagnetic materials have a significant relaxation time, which is the time required for their magnetic dipoles to realign after the removal of an external field, and the magnetic susceptibility of paramagnetic materials is thus frequency-dependent. In alternating fields, especially at high frequencies, the direction of the field changes faster than the dipoles can realign, preventing an effective response, especially if their magnetic moments become “pinned” in the direction of a simultaneously applied static field [50].

The Earth’s magnetic field, although relatively weak compared to artificial magnets, extends far into space and can influence the motion of charged particles and magnetic materials. This interaction leads to the formation of the Van Allen radiation belts around the Earth and moves the compass arrow. Geomagnetic cues (inclination, declination, and total Earth’s magnetic field intensity) provide spatial gradients across the globe and could be used for navigation. Can this field affect paramagnetic or even superparamagnetic melanosomes or affect their movement in microvilli?

## 4. Why the Retina May Be Involved in Magnetic Sensing

It has been shown that, behaviorally, many animals possess sensitivity to magnetic fields and use them for navigation tasks ranging from local homing to long-distance migration [51]. Speaking of vertebrates, different hypotheses of magnetoreception have been proposed, including one proposing a chemical compass based on a chemical radical pair mechanism situated in short-wavelength (blue light) sensitive cryptochrome photoreceptors and the other postulating processes involving magnetite particles [51]. In birds, magnetite particles were proposed to localize in the beak (inside special neurons), which may drive magnetoreception [52,53]. Later, it was shown that there is no magnetite in the neurons in the beak, and previous data were just dried macrophages [54]. There is also evidence of changes in retinomotor movements under a small magnetic field [55]. The majority of these ideas have the retina as the main magnet-sensing organ. Here we will discuss these ideas sequentially.

While many animals supposedly have magnetic sensitivity, avian magnetic sensitivity has rock-solid evidence [52,56]. Of course, birds have other compasses as well, like the sun and polarized light, the stars and their constellations, and the geomagnetic field is only one part of the orientation system [57]. Passerine long-distance migrant birds like the Pied flycatcher (Ficedula hypoleuca, see Figure 1) can sense both the intensity and the inclination (angle) of the Earth’s magnetic field but are not responsive to the north–south direction [52]. Because birds’ orientation in vivo functions properly under short-wavelength but not long-wavelength visible light, their magnetic sensitivity is suggested to be light- and wavelength-dependent and localized in the eyes [52,58]. There is also some evidence from in vitro experiments. It was shown that magnetic field direction changes induce significant effects on electroretinogram (ERG) amplitude in response to low-intensity blue flashes in the nasal region of the isolated retina of pigeons and robins, while exaggeratedly bright blue flashes reduce the response, which implies the participation of light adaptation mechanisms in magnetic sensitivity [59]. ERG also responded to red flashes in the presence of constant low-intensity blue light. The nasal/central retina presents a higher density of double photoreceptor cones in birds and is particularly sensitive to short-wavelength light that also allows higher resolution [26,60]. Double cones of several bird species, including chicken, express cryptochrome 4, which could be the primary magnetic sensory molecule as it binds the crucial FAD (flavin adenine dinucleotide) cofactor [61]. As signal proteins related to DNA photolyases, cryptochromes (CRYs) play a key role in short-wavelength photo-sensing, perception of the magnetic field, and circadian rhythm entrainment in some animals [62].

Cryptochromes and their effects: Observations have shown that plants respond to magnetic fields. One of the earliest and most significant pieces of evidence implicating plant cryptochrome blue light receptor molecules [63]. Research demonstrated that a static magnetic field of about 10 times the Earth’s magnetic field reduced the growth of seedlings in blue light [64,65]. This effect was observed under blue/UV light but not in red light, and it was absent in seedlings that were cryptochrome mutants, indicating a role for cryptochromes in the response. Subsequent studies confirmed the involvement of both Cry1 and Cry2 in modulating blue-light-dependent plant growth responses to static magnetic fields [66,67].

Similarly, cryptochromes could function as magnetoreceptors in migratory birds. Behavioral and histochemical studies revealed a connection between light sensitivity and the role of cryptochrome 1a in the retina of birds [51].

It was also shown that purified night-migrating Robin cryptochrome 4 (Cry4) has significant magnetic sensitivity (30 mT magnetic fields changed the absorption of FAD after blue photoexcitation (450 nm)). The magnetic sensitivity of Cry4 from the night-migratory robin was substantially larger than that of the Cry4 proteins from the non-migratory—primarily diurnal—pigeon and chicken [68]. Interestingly, the authors also say that Cry4 molecules are not expected to show responses to Earth-strength magnetic fields around 50 μT in vitro. To get a response requires restricting the internal mobility of the radicals. The authors suggested that to determine whether Cry4 acts as a magnetoreceptor molecule in vivo, direct manipulations of this protein in the eyes of night-migratory songbirds would be required.

Retinomotor movement and magnetic influence: Retinomotor reaction refers to the movement of photoreceptors and melanosomes in RPE in the retina in response to changes in light conditions. This reaction involves the elongation and contraction of photoreceptor cells, specifically cones and rods, and the movement of melanosomes inside RPE microvilli related to light adaptation. These movements help optimize retinal light sensitivity and visual acuity. Some authors have shown that cones and rod elongation can be affected by magnetic fields [55,69,70]. While the concept of retinomotor reactions is well documented in teleosts (fish), studies on birds and other vertebrates indicate that they also possess similar mechanisms, although the specifics may vary. In the teleost fish retina, retinomotor movements are characterized by the following:

### In Darkness:

Cones elongate until the RPE, while rods contract completely, and pigment granules from microvilli aggregate at the cell body of the RPE cell. Cone elongation is induced when light-adapted retinas are exposed to exogenous cAMP or dopamine [71]. The motile cone fragments consist of both inner and outer segments (CIS-COS, so-called ellipsoid). The principal mechanism of elongation is, most probably, the cytoskeletal myeloid. Isolated cone ellipsoids possess sufficient cytoskeletal and regulatory machinery to exhibit light- and dopamine-regulation retinomotor movement similar to that observed in intact cones in situ [72]. This adaptation enhances sensitivity to low light levels (scotopic vision).

### In Twilight:

During twilight, both rods and cones are activated. Rods are more sensitive to low light levels, while cones are responsible for color vision and function best in brighter light. In the twilight phase, the retina utilizes a combination of these photoreceptors to optimize so-called mesopic vision. In these conditions, the length of the rod and cone became intermedial. In normal twilight conditions, the nuclei of the rod cells are positioned just above the external limiting layer of the retina, while cones are partially displaced closer to the pigment epithelium, but not until the RPE cell body.

### In Light:

The movements reverse—cones completely shorten and rods expand until the RPE cell bodies, RPE microvilli, become filled with melanosomes, allowing for better performance and acuity under bright conditions. Interestingly, deep red light is less effective in producing light adaptation than blue light [73].

Cone movements are regulated by various biochemical signals, including cyclic adenosine monophosphate (cAMP), which has been shown to induce cone elongation even under bright light conditions. The levels of cAMP are typically higher in dark-adapted retinas compared to light-adapted ones, suggesting a role in the circadian regulation of these movements [71]. The magnetic field was shown to affect retinomotor reactions.

The magnetic field affects cone–rod elongation. Retina photoreceptors of the salmon Oncorhynchus masou exposed to a constant magnetic field (80 Gs) under conditions of twilight illumination demonstrated not a twilight reaction, as in the control, but a darkness (scotopic) reaction. The RPE in this case reacted, on the contrary, as to bright light. After the application of long-wavelength red light, the retinomotor reaction returned to partial light adaptation [72]. Also, in some animals, the compensation of external Earth’s geomagnetic field (GMF) in twilight instead of normal elongation of cones produces a very extensive elongation of mainly double and central cones, while rods are not shortened enough, only vaguely corresponding to a reaction to mesopic adaptation [69]. A very interesting retinomotor reaction is described in migrating salmon species and artificially produced dwarf salmon under conditions of normal light. In migratory salmon, rods and double cones perceived neutralization of the GMF as the onset of darkness (the scotopic reaction), while single (generally blue-sensitive) cones responded to neutralization of the GMF as a presentation of blue light. The neutralization of the GMF rods of dwarf animals showed a light (photopic) response, while double (red/green-sensitive) cones produced dark (scotopic) responses. Single (blue-sensitive) cones responded to the neutralization of the GMF as bright-blue light, similar to migrating animals. Thus, the morphological picture of the retina in salmon under these experimental conditions corresponds to the perception of blue light [70].

While the cryptochromes hypothesis of magnetic sensitivity is the most developed one, the following questions arise:

- Generally, the involvement of such different cells in the retina in motor reactions to magnetic fields suggests that there may be different receptors of the magnetic field because cryptochromes are found in only one particular type of photoreceptor.
- Also, it was found that the optical branch of the trigeminal nerve is necessary for the perception of the magnetic map in birds, and its denervation disrupts magnetic sensing [57,74]. Anatomically, the ophthalmic branch of the trigeminal nerve (V1) (so-called long ciliary nerve) does provide sensory innervation to various structures around the eyes, including the cornea, conjunctiva, the lens, and ciliary body, with the areas working together to get light to the right spot on the retina. This sensory branch also directly innervates the choroid [75,76]. While it is not known if RPE cells are innervated by V1, it was shown that sensory nerves in the choroid occur mainly alongside the Bruch membrane near RPE [75,77]. If RPE cells’ metabolic needs are supported indirectly through the innervation of the choroid, they may also be affected after denervation. Are RPE cells directly innervated by this nerve and involved in magnetic sensitivity?

On the other hand, magnetic response in the isolated retina has shown that the feedback from RPE through the trigeminal nerve is not necessary for some magnetic sensing, or there may be many magnetic sensors working in parallel.

- It is known that RPE cells hyperpolarize during the light adaptation cycle and depolarize in darkness, generating a transepithelial potential and contributing to the direct current electroretinogram c-wave (RPG) recorded as a multi-faze slow component of RPG, with a peak during light adaptation [23,78]. It is known that the greatest magnetic effect on RPG occurs during the light cycle [59]. Are RPE cells involved in generating the magnetic-sensing response on electroretinograms (RPG)?
- There are currently no data on how cryptochrome’s response to the magnetic field is transduced to RPG changes and converted to cell movements in the retina.
- How can cryptochrome’s blue-light sensitivity explain that high-intensity blue flashes reduce retinal responses to magnetic field switches [59]? Why do red-light flashes induce responses to magnetic field switches in twilight?
- Cryptochrome’s magnetic sensitivity in both plants and the retina is only observed in a relatively big magnetic field (50 times that of the Earth’s).

Interestingly, one study indicated that variations in low magnetic field strength can significantly impact the enzyme hydroxy indol-O-methyl transferase (HIOMT), which is involved in light adaptation processes within the retina and pineal gland in birds and humans [79]. This may be another hypothesis of magnetic sensing.

## 5. Can Modern Physics Support the Melanin Magneto-Optical Sensing Hypothesis?

While the Earth’s magnetic field can align the magnetic moments of superparamagnetic particles, it is generally too weak and uniform to move them significantly without the presence of a strong magnetic gradient. Maybe melanosomes train (chain) can be more responsive? Under extreme light conditions, the melanosome chain is fully present in the microvilli (light adaptation). For paramagnetic particles, especially those with superparamagnetic properties, the application of a magnetic field can induce magnetization and cause the particles to aggregate into chains. However, their movement under a magnetic field involves a balance between magnetic forces and other forces such as viscous forces. For instance, a rotating magnetic field can induce the formation of vortices around rotating chains of paramagnetic particles, which can then move in a controlled manner [65,79]. However, this typically requires a magnetic field of sufficient strength and frequency to overcome the opposing forces. Also, as with the cryptochrome hypothesis, there are no data on how particle movements are transduced to electric potentials and other responses.

## 6. Conclusions

While several hypotheses exist regarding magnetic sensing, the most detailed one involves cryptochrome, although it still has numerous unclear aspects. The melanin magnetic particles hypothesis, despite its apparent simplicity, requires further experiments and evidence to be fully validated.

## Data Availability

No new data were created or analyzed in this study. Data sharing is not applicable to this article.
